# Relationship between executive function and activities of daily living in Alzheimer’s disease: a study based on the stop-signal task

**DOI:** 10.3389/fnagi.2026.1837948

**Published:** 2026-07-10

**Authors:** Manman Gao, Yibing Yan, Yue Wu, Zhi Geng, Lu Wang, Shanshan Zhou, Panpan Hu, Xingqi Wu, Kai Wang

**Affiliations:** 1 Department of Neurology, The First Affiliated Hospital of Anhui Medical University, Hefei, China; 2 Anhui Province Key Laboratory of Cognition and Neuropsychiatric Disorders, Hefei, China; 3 Collaborative Innovation Center of Neuropsychiatric Disorders and Mental Health, Hefei, China; 4 Department of Neurology, The Central Hospital of Wuhan, Tongji Medical College, Huazhong University of Science and Technology, Wuhan, China; 5 Department of Psychology and Sleep Medicine, The Second Affiliated Hospital of Anhui Medical University, Hefei, China; 6 Institute of Artificial Intelligence, Hefei Comprehensive National Science Center, Hefei, China; 7 School of Mental Health and Psychological Sciences, Anhui Medical University, Hefei, China

**Keywords:** activity of daily living, Alzheimer’s disease, event-related potentials, executive function, stop-signal task

## Abstract

**Background:**

Early clinical manifestations of Alzheimer’s disease (AD) include an apparent decline in memory and executive function. Executive function is closely related to activities of daily living (ADL) and is important for maintaining an independent, high-quality lifestyle.

**Objective:**

This study aimed to explore the executive control ability and neuromechanisms of AD patients through stop-signal task (SST) elicited event-related potentials (ERPs) and their relationship with ADL.

**Methods:**

Thirty-six patients with AD and 36 sex and age matched healthy controls (HCs) were recruited. Electroencephalography (EEG) data recorded during the SST was compared between groups, and SST-related indicators were determined to assess executive control ability in AD patients. The relationship between ADL and SST-related indicators was explored. We performed Receiver Operating Characteristic (ROC) analysis on SST- and EEG-related indices.

**Results:**

Differences in the following indices were found between the two groups: Go accuracy (*P*< 0.001), Go omissions (*P*< 0.001), Go errors (*P*< 0.001), Go error reaction time (RT) (*P*< 0.001), failed stop RT (*P* = 0.021), all accuracies (*P* = 0.005), mean amplitude of N300 (*P* = 0.043), peak amplitude of N300 (*P* = 0.043), and peak latency of N300 (*P*< 0.001). And Go accuracy (*r* = −0.603, *P* = 0.005) and all accuracy (*r* = −0.624, *P* = 0.003) in the AD group were negatively partially correlated with ADL. These SST- and EEG-related indicators had an Area Under the Curve of 0.771 and 0.831, both of which could be used to jointly diagnose AD (both *P*< 0.001).

**Conclusion:**

This study suggests that the worse the executive function of AD patients, the more serious the ADL impairment. AD patients have electrical abnormalities associated with executive control. Different SST- and EEG-related indicators can be used to diagnose AD. This provides a new avenue for further elucidation of the pathological mechanisms of AD.

## 1 Introduction

Alzheimer’s disease (AD) is the most common form of dementia, and as the condition progresses, patients’ memory, thinking, and language skills gradually decline, limiting their activities of daily living (ADL) (Lane et al., 2018). This brings significant challenges to patients and their caregivers (Cerejeira et al., 2012; Song and Oh, 2015), and is becoming an increasingly severe global health problem. Studies have shown that executive function is closely related to ADLs, and may be more important than memory when it comes to the ability to maintain an independent high-quality of life (Alzheimer’s Dementia, 2022; Collette et al., 1999).

In addition to obvious memory decline, early clinical manifestations of AD include deterioration of executive function (such as manipulation, switching, monitoring, and inhibition), which may occur earlier than memory decline (Balota et al., 2010; Hazlett et al., 2015). Executive function is a collection of higher-order cognitive processes that organize and adapt goal-oriented behaviors, including response inhibition, task switching, and working memory updating (Munakata et al., 2011). Response inhibition refers to the inhibition of the action-response impulse, which is a key component of executive control. Specifically, response inhibition refers to the inhibition of unnecessary or inappropriate behaviors so that people can carry out various flexible and purposeful behavioral responses to the external environment (Huster et al., 2020; Yeung et al., 2021). Response inhibition is a complex attention control process with the advantage of a high response and is considered the foundation on which other cognitive domains function (Yeung et al., 2021).

Few studies have investigated the function of response inhibition in patients with AD. The two most common behavioral tasks used to study inhibition processing are Go/No-Go and stop-signal tasks (SSTs) (Camfield et al., 2018). In recent years, the SST has been mainly used in the fields of developmental psychology, neuropschiatry, and cognitive neuroscience. In developmental psychology, researchers have found that children and older people have longer stop-signal reaction times (SSRTs) than adults. A comparative study of the SSRT and Go reaction time (Go RT) also found that the development and decline of general reaction and inhibition abilities were independent processess (van den Wildenberg and van der Molen, 2004). In neuropsychiatry, the SST has been widely used to study inhibitory function impairment in schizophrenia, obsessive-compulsive disorder, multiple tic disorder, Parkinson’s disease (PD), and other diseases. The SSRT of patients with schizophrenia is significantly higher than that of healthy individuals (Enticott et al., 2008; Rush et al., 2006). The SST has also been used in cognitive neuroscience to study individual inhibitory functions and the underlying neurophysiological mechanisms of neural abnormalities. For example, studies have shown that the frontal lobe and pre-auxiliary motor area are involved in response inhibition (Harvey and Lepage, 2007; Hsieh and Lin, 2017).

At present, the SST and its corresponding response inhibition model are still being developed and improved, and it is predicted that this research tool will be increasingly applied to study the underlying mechanisms of cognition and the nature of clinical psychiatric diseases. However, whether abnormal response inhibition occurs in patients with AD and the underlying mechanisms remain unknown.

Event-related potentials (ERPs) are widely used in the study of cognitive processes and diseases, and electrophysiological studies of response suppression have shown that No-Go N2 (200–400 ms) and No-Go P3 (300–600 ms) ERPs in the mid-frontal line most commonly occur at the same time as No-Go or No-Stop stimulation (Huster et al., 2013). In the SST, the N200 and P300 latencies were slowed, and the amplitude was generally reduced in the elderly compared to the young. This is consistent with previous conclusions regarding cognitive aging (Elverman et al., 2021). A significant error-related negativity (ERN) component was observed in both the high trait anxiety (HTA) and low trait anxiety (LTA) groups, and the mean amplitude was significantly reduced in the HTA group compared to the LTA group, suggesting that errors were detected more quickly and efficiently in patients with LTA. There was no significant difference in the peak latency between the groups, suggesting that trait anxiety had no substantial effect on response inhibition ability (Hsieh et al., 2021). In a study of local field potentials in patients with PD, it was found that the subthalamic nucleus is involved in successful inhibition in the SST (Alegre et al., 2013). Although ERPs may be sensitive markers of emerging AD-related neurological dysfunction (Green et al., 2015), further study is required to test its diagnostic ability. Therefore, we speculate that the SST can also be used as an indicator of disease differentiation and prediction of disease progression in patients with AD.

This study used ERPs elicited in the SST to determine executive control ability and its potential neural mechanisms in patients with AD and explored the relationship between them and impaired ADL. We hypothesized that: (1) the response inhibition ability of AD patients is impaired, which manifests as the deterioration of SST performance; (2) there are changes in executive control-related brain activity in AD patients; (3) deterioration of SST performance in patients with AD is partially correlated with ADL impairment; and (4) different SST- and EEG-related indicators can be used to diagnose AD.

## 2 Materials and methods

### 2.1 Participants

We recruited thirty-six patients with AD from the First Affiliated Hospital of Anhui Medical University, China, between November 2021 and March 2023. Patients with AD were clinically diagnosed by a specialist according to the National Institute of Neurological and Communicative Disorders and Stroke and the Alzheimer’s Disease and Related Disorders Association (NINCDS-ADRDA) criteria (McKhann et al., 1984) as follows: (a) the criteria for possible or probable AD were met; (b) Mini-Mental State Examination (MMSE) scores were < 24; and (c) Clinical Dementia Rating (CDR) scores ranged from 0.5–2. Patients with substance use disorders, other neurological diseases, and life-threatening somatic diseases were excluded. Thirty-six age- and sex-matched healthy controls (HCs) were recruited from the local community through advertisements or the spouses of study participants. The inclusion criteria for HCs were: (1) normal cognitive function; (2) no neurological or psychiatric disorders; (3) no previous psychoactive medication use; (4) MMSE scores ≥ 27; and (5) a Clinical Dementia Rating (CDR) score equivalent to 0. All participants had normal visual acuity or corrected visual acuity without visual impairment, and all participants were right-handed and provided written informed consent. The study was conducted in accordance with the latest revision of the Declaration of Helsinki, and the local ethics committee of Anhui Medical University approved the experimental procedures. Sample size calculation of this study referred to the methods of previous clinical studies (Serdar et al., 2021; Wang and Ji, 2020) . The significance level was set at α = 0.05 and statistical power at 1-β = 0.8. The minimum sample size was estimated according to the expected effect size of primary outcome indicators. With an additional 15% reserved for loss to follow-up and invalid cases, the final enrolled sample size is sufficient to meet the requirements for statistical analysis.

### 2.2 Neuropsychological assessment

All of our participants were assessed with clinical and neuropsychological tests. The neuropsychological tests we conducted included: (i) the MMSE test and Montreal Cognitive Assessment–Beijing Version (MoCA) were used to assess participants’ general cognitive function; (ii) the Lawton-Brody Activities of Daily Living (ADL) scale was used to assess participants’ daily functions; and (iii) the CDR was used to assess the severity of the disease. All of these tests contributed to our clinical diagnosis of AD.

### 2.3 Experimental task

#### 2.3.1 The SST

**FIGURE 1:**
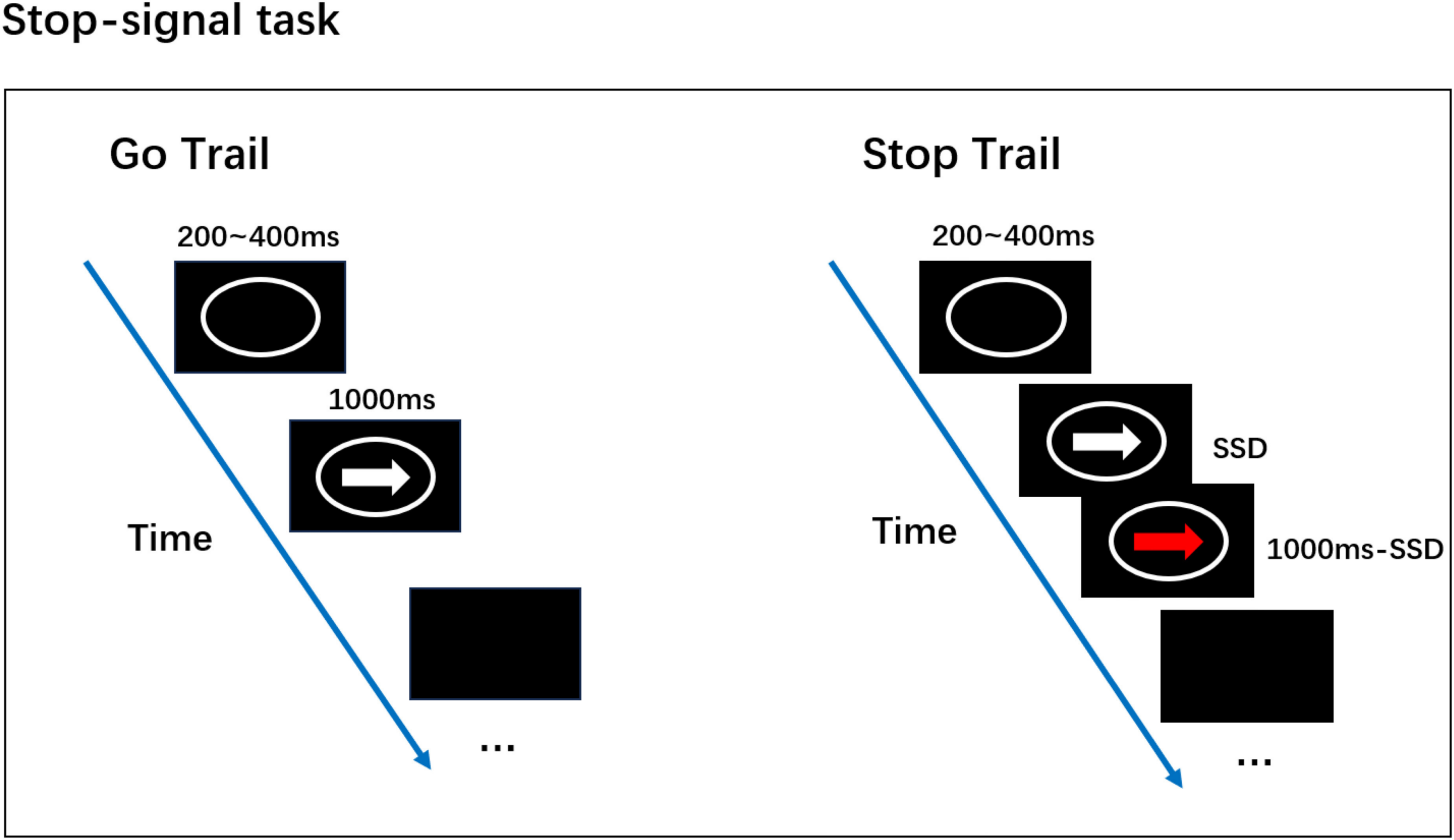
The stop-signal task. Timeline of Go and Stop trials in Stop-signal task. Arrows to the left and right appear in the Go trail (reaction signal), and Stop signal (red arrow) appear in the Stop trail in addition to the reaction signal. SD, Stop signal delay time.

EEG data were recorded while the subjects participated in the SST (Figure 1). E-Prime Software (Psychology), a software tool programmed for the current study, was adapted from the study by Yu et al. (2022). During the experiment, the influence of external interference factors such as sound and light was excluded by providing the subjects with a quiet and comfortable experimental environment. Experimental tasks were presented using E-Prime on a computer with a screen size of 21.5 inches. The experimental task consisted of two types of signals: a reaction signal (Go signal) and a stop signal (Stop signal), which were presented at a ratio of 70:30. The computer presented two signals randomly, totaling 200 trials. Before the experiment began, the participants were given clear instructions and practiced the task to become fully familiarized with its content. Initially, a white fixation point in the shape of a circle was presented in the center of the black screen for 200–400 ms, and then a white arrow appeared in the circle (facing left or right). In the Go signal, upon seeing the white arrow, the subjects were asked to make a quick and correct key response (by pressing the “F” key when the arrow was facing left, press the “J” key when it faced to the right), but some white arrows suddenly turned red at an unpredictable timepoint, and this red arrow was the Stop signal. When the arrow turned red, the participants had to restrain their impulses to press the button. The Go task had only a Go signal and its presentation time was 1,000 ms. In the SST, the Go signal is presented first, followed by a random stop signal. The time interval between the Go signal and Stop signal is called the stop-signal delay (SSD) time; therefore (1,000-SSD) ms is the stop-signal presentation time. The initial SSD was set to 250 ms in our task and the step size was 50 ms. If the participant failed to suppress their response in the Stop trial, the SSD was progressively reduced by 50 ms until it reached 0 ms. Conversely, if the participant successfully suppressed the response to the Stop signal, the SSD was increased gradually until it reached 550 ms. During the entire experiment, when explaining the requirements of the experimental task to the subjects, the experimenter specifically pointed out that the subjects should not deliberately wait for the arrow to turn red, because the time for each arrow to turn red was insufficient. At the same time, the experimenter also told the subjects that it was normal for them to fail to restrain the impulse to press the buttons successfully. Therefore, there was no need to deliberately adjust or question the reaction mode. Subjects were asked to try to keep their mind and body relaxed.

#### 2.3.2 Calculation of SST-related indicators

Variables of interest included the Go accuracy, omissions and errors, average Go RT, the stop accuracy, mean SSD, average RT on unsuccessful Stop trials, and SSRTs. We calculated the accuracy, omission, and error rates of each subject’s Go Signal and the average response time of the correct and wrong Go signals. The accuracy rate of each subject’s Stop signal, mean response time of error, mean SSD, mean SSRT, and overall accuracy rate of both signals were also calculated. The SSRT is a critical behavioral indicator of Stop-signal tasks. The SSRT is equal to the mean Go signal response time minus the stop-signal delay time (mean SSRT = mean Go RT-mean SSD). The SSRT refers to the subject’s reaction time in successfully suppressing an internal action impulse, that is, the time spent from the Stop signal’s appearance to the Stop task’s completion, which can effectively reflect the response speed of the subject to the Stop signal. The higher the SSRT, the longer the subject’s response time to the Stop signal, and the worse the response inhibition ability; the lower the SSRT, the more quickly the subject can suppress the reaction impulse, and the better the response inhibition ability (Yu et al., 2022). Participants were excluded if the mean RT of correct or incorrect failed Stops (failed Stops where the keypress did or did not accord with the stimulus) was more significant than the mean Go RT (Verbruggen et al., 2019).

### 2.4 EEG data acquisition and processing

#### 2.4.1 EEG data acquisition

This experiment was performed using an extended international 10/20 system (Waveguard64 cap, Cephalon A/S) using 64 Ag/AgCl electrodes placed on the scalp for electroencephalography (EEG) recording. Before recording the EEG, we asked the subjects to wash their scalp to avoid affecting the contact effect between the electrodes and the scalp, so as to monitor the EEG activity of the cerebral cortex more accurately. The left upper and lower vertical electroophthalmograms (EOGs) and the left and right orbital margin horizontal EOGs were recorded, respectively. EEG activity was recorded using the left mastoid reference electrode, and the mean value of both mastoid electrodes was referenced offline. The impedance of all electrodes in this experiment is below 5 kΩ. EEG and EOG activities are amplified with DC 0.01–100 Hz band-pass and the continuous sampling rate is 512 Hz (64-channel high speed amplifier, Advanced Neuro Technology, Enschede, Netherlands).

#### 2.4.2 EEG data processing

**FIGURE 2:**
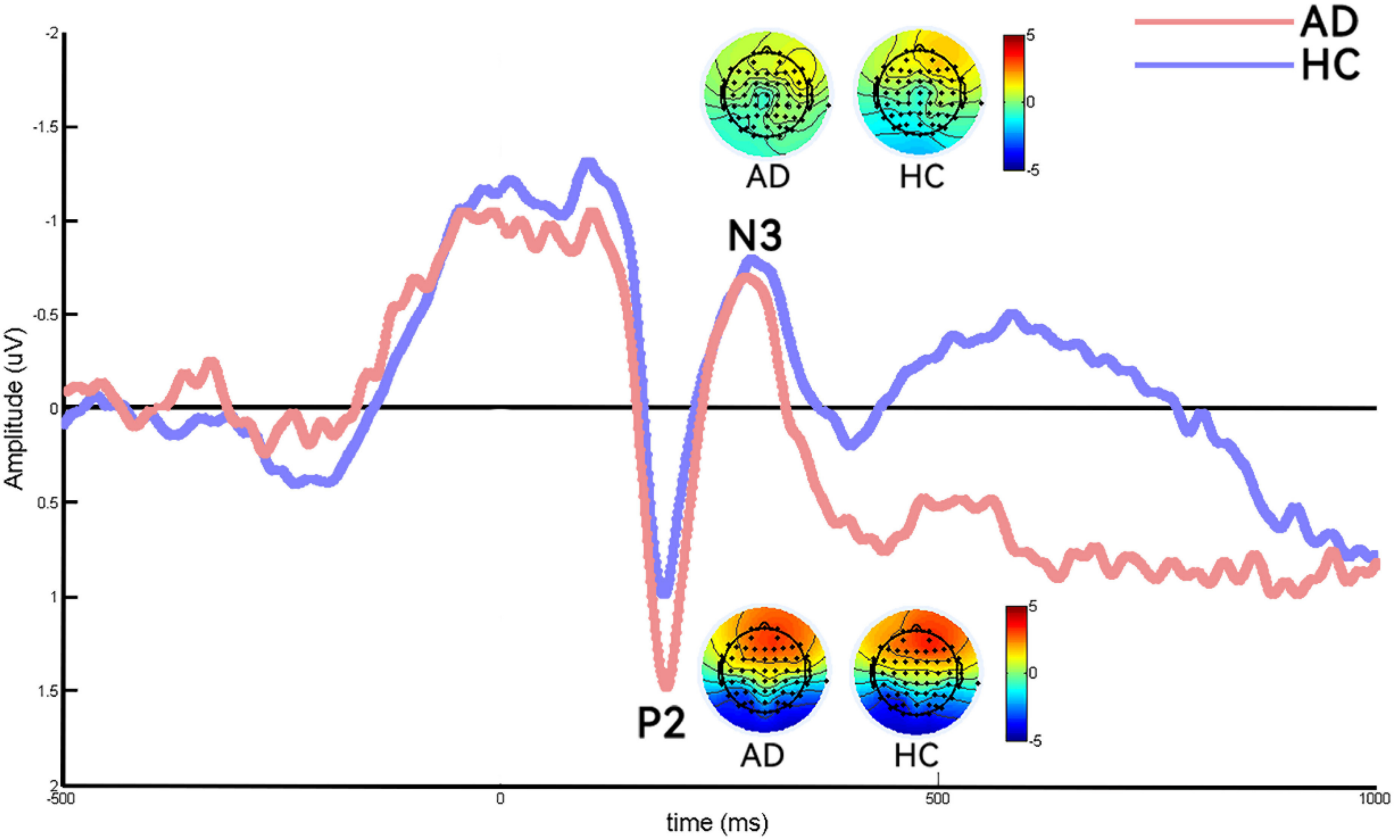
Waveform maps of AD and HC groups in CZ channel and topographic maps of N3 and P2 points. Red represents the waveform diagram of AD group and blue represents the waveform diagram of HC group. Topographic maps of AD/HC group at N300 and P200 are drawn, respectively. An increase in spectral power is illustrated by warm colors, a decrease in spectral power by cool colors. AD, Alzheimer’s disease; HC, healthy controls; N3, N300 (250~350 ms), P2, P200 (150~250 ms).

First, the collected EEG data were processed using MATLAB and EEGLAB toolkits. The data were processed according to the following steps: (1) channel positioning; (2) removal of useless electrodes; (3) filtering; (4) sampling rate reduction; (5) segmentation, baseline correction, removal of the bad segments, and replacement of the bad electrode; (6) independent component analysis (ICA) (Palmer et al., 2008); (7) manual check of the independent components to find and remove the artifact component; (8) use of the extreme value method to remove artifacts; (9) re-reference (whole brain average reference); (10) manual browsing of the data to ensure that there were no other artifacts; and (11) saving the data to complete the data preprocessing (Rodrigues et al., 2021). Based on evidence from previous studies of time-frequency graphs, θ activity was found to be strongest in SST tasks and most prominent at the midline (Cz electrode site) above the central frontal region (Andrews et al., 2023). Therefore, the Cz channel was selected as the channel of interest, and its waveform and topographic maps were drawn (Figure 2). The processed ERP waveforms were locked for 500 ms before and 1,000 ms after the signal. Then, the peak values of P200 (150 250 ms), N300 (250 350 ms) and other EEG-related indices, such as the mean amplitude and peak amplitude of N300, the mean amplitude and peak amplitude of P200, and peak latency of P200, were extracted.

### 2.5 Statistical analyses

All data were analyzed using SPSS software (version 20.0; SPSS Inc., Chicago, Illinois, United States). Differences in demographic and clinical characteristics between the AD and HC groups were assessed using the chi-square test, two-independent sample *t*-test, or Mann-Whitney U-test. As the data did not follow a normal distribution, we used Mann-Whitney U-tests to evaluate the differences in relevant indicators between the AD and HC groups when participating in the SST. At the electrophysiological level, we also conducted Mann-Whitney U-tests on the extracted EEG indicators to evaluate the EEG differences between the AD and HC groups during the SST. Since the gender and age of subjects in the AD and HC groups were matched, the years of education of the two groups were different. Considering the effects of age and years of education on executive control tasks and EEG (Kaszniak et al., 1979), they were correlated as covariates, and SST-related indices and EEG-related indices of AD and HC were correlated with ADLs. In the correlation analysis, age and years of education were included as covariates. Since some indicators did not follow a normal distribution, Spearman’s rank correlation analysis was adopted. Correlation coefficients (r) and two-tailed *p*-values are reported. We performed Receiver Operating Characteristic (ROC) analysis on SST-related and EEG-related indices. The ROC curve was plotted with sensitivity on the *y*-axis and 1-specificity on the *x*-axis. The area under the curve (AUC) and its 95% confidence interval (95% CI) were calculated to evaluate the discriminative ability of the index. Meanwhile, the optimal cut-off value was determined using the Youden’s index (Youden’s index = sensitivity + specificity - 1) to balance sensitivity and specificity. All statistical analyses were performed using SPSS 20.0 software, and a two-tailed *P*-value < 0.001 was considered statistically significant.

## 3 Results

### 3.1 Sample demographic and clinical characteristics in each diagnostic group

There were no significant differences in age or sex between the AD and HC groups; however, there were differences in the years of education (*P*< 0.001), MMSE scores (*P*< 0.001), MoCA scores (*P*< 0.001), ADL (*P*< 0.001), and CDR (*P*< 0.001) (Table 1).

**TABLE 1:** Sample demographic and clinical characteristics in AD and HC groups.

Variable	AD (*N* = 36)	HC (*N* = 36)	T/Z/χ ^2^	*P*
Sex (M/F) ^a^	18/18	13/23	1.416	0.234
Age (years) ^b^	61.14 ± 8.695	62.32 ± 7.377	−0.590	0.557
Education (years) ^b^	8.486 ± 4.6550	12.414 ± 2.6494	−4.032	<0.001
MMSE ^c^	17.87 ± 4.299	28.46 ± 1.401	−6.131	<0.001
MoCA ^c^	15.24 ± 6.286	26.11 ± 2.079	−6.478	<0.001
ADL ^c^	25.79 ± 5.433	20	−7.038	<0.001
CDR ^c^	0.68 ± 0.638	0	−4.844	<0.001

^a^Chi-square test.

^b^Independent two-sample *t*-test.

^c^Mann–Whitney U-tests. AD, Alzheimer’s disease; HC, healthy controls; M, male; F, female; MMSE, Mini Mental State Examination; MoCA, Montreal Cognitive Assessment- Beijing Version; ADL, Activities of Daily Living; CDR, Clinical Dementia Rating.

### 3.2 Differences of SST-related indexes and EEG-related indexes between the AD and HC groups

**FIGURE 3:**
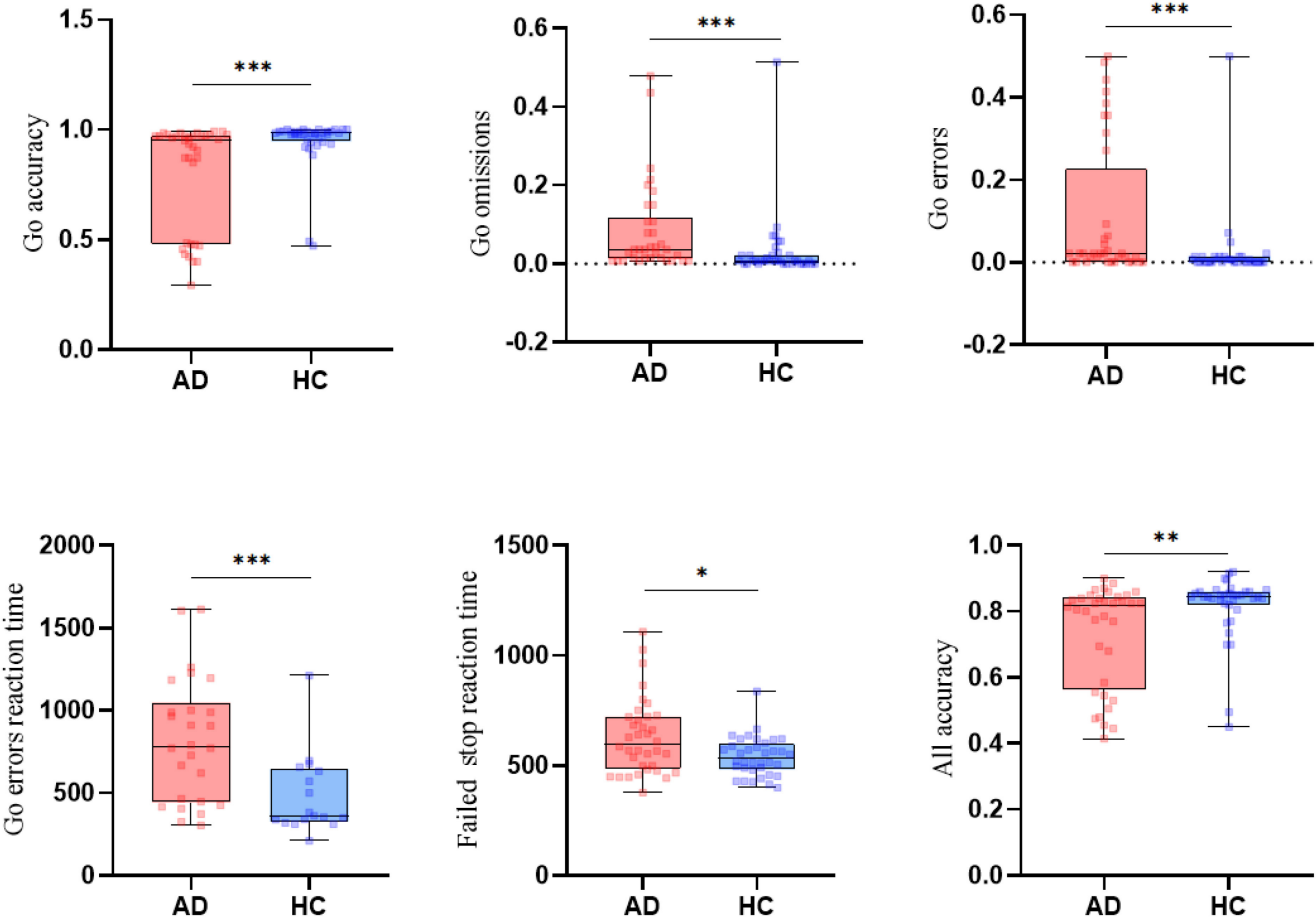
Differences of SST-related indexes between AD and HC groups. Go accuracy, Go omissions, Go errors, Go errors reaction time, Failed stop reaction time and All accuracy showed differences in AD and HC groups, and these differences were statistically significant. AD, Alzheimer’s disease; HC, healthy controls. *p< 0.05, **p< 0.01, ***p< 0.001.

**FIGURE 4:**
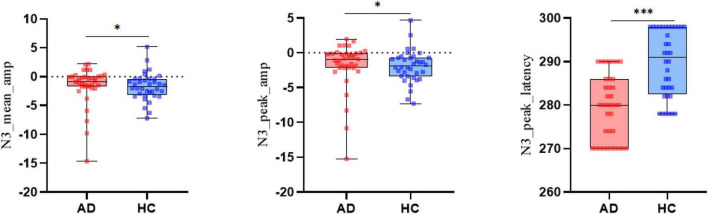
The difference of EEG-related indexes between AD and HC groups. Mean amplitude of N300, peak amplitude of N300, and peak latency of N300 are different in AD and HC groups, and these differences were statistically significant. N3_mean_amp, mean amplitude of N300; N3_peak_amp, peak amplitude of N300; N3_peak_latency, peak latency of N300. *p< 0.05, ***p< 0.001.

Table 2 shows the descriptive statistical results of-related and EEG-related indices and the results of the Mann-Whitney U-tests when the AD and HC groups participated in the SST. The following SST-related indices, Go accuracy (*P*< 0.001), Go omissions (*P*< 0.001), Go errors (*P*< 0.001), Go error reaction time (*P*< 0.001), failed Stop reaction time (*P* = 0.021), and all accuracies (*P* = 0.005), differed between the AD and HC groups (Figure 3). The differences in EEG-related indicators are shown in Figure 4, where the mean amplitude of the N300 (*P* = 0.043), peak amplitude of the N300 (*P* = 0.043), and peak latency of the N300 (*P*< 0.001) differed between the AD and HC groups.

**TABLE 2:** Differences of SST-related indexes and EEG-related indexes between AD and HC groups.

Related indexes	AD (*N* = 36)				HC (*N* = 36)				*Z*	*P*
	*M*	SD	Min	Max	*M*	SD	Min	Max		
Go accuracy	0.807	0.241	0.293	0.993	0.947	0.118	0.471	1	−3.968	<0.001
Go omissions	0.085	0.116	0.007	0.479	0.031	0.086	0.000	0.514	−3.862	<0.001
Go errors	0.112	0.169	0.000	0.500	0.023	0.086	0.000	0.500	−3.347	<0.001
Go errors RT	821.792	377.020	303.500	1613.068	483.694	241.911	210.000	1212.000	−3.204	<0.001
Go RT	707.358	210.469	443.871	1480.907	633.958	135.663	202.914	865.500	−0.935	0.350
Stop accuracy	0.558	0.162	0.017	0.850	0.498	0.181	0.000	0.767	−0.949	0.343
Failed stop RT	630.316	171.196	376.567	1107.526	540.064	88.362	399.367	837.034	−2.309	0.021
SSD	345.069	141.632	65.833	520.833	320.116	126.167	58.333	496.667	−1.154	0.248
SSRT	362.288	159.280	64.517	970.907	313.843	57.547	107.081	419.667	−0.788	0.430
All accuracy	0.733	0.155	0.415	0.900	0.817	0.098	0.450	0.920	−2.819	0.005
N3_mean_amp	−1.592	3.335	−14.663	2.189	−1.933	2.176	−7.133	4.626	−2.027	0.043
N3_peak_amp	−1.849	3.460	−15.248	1.970	−2.149	2.179	−7.242	4.249	−2.027	0.043
N3_peak_latency	279.330	7.797	270.000	290.000	299.610	8.813	290.000	310.000	−6.862	< 0.001
P2_mean_amp	1.058	1.876	−4.625	4.221	0.752	1.950	−1.823	7.904	−1.470	0.142
P2_peak_amp	1.448	1.961	−4.369	4.974	1.141	1.967	−1.746	8.032	−1.216	0.224
P2_peak_latency	188.330	7.612	176.000	196.000	186.940	7.099	176.000	196.000	−0.897	0.370

AD, Alzheimer’s disease; HC, healthy controls; RT, reaction time; SSD, Stop signal delay; SSRT, Stop signal reaction time; stop signal reaction time = go reaction time-stop signal delay; N3_mean_amp: mean amplitude of N300; N3_peak_amp: peak amplitude of N300; N3_peak_latency: peak latency of N300;P2_mean_amp: mean amplitude of P200; P2_peak_amp: peak amplitude of P200; P2_peak_latency: peak latency of P200.

### 3.3 Correlation analyses

Table 3 shows that, after controlling for the influence of age and years of education, Go accuracy (*r* = −0.603, *P* = 0.005) and All accuracy (*r* = −0.624, *P* = 0.003) in the AD group were negatively correlated with ADL. Go errors (*r* = 0.544, *P* = 0.013), Go errors RT (*r* = 0.573, *P* = 0.008), and failed stop (*r* = 0.488, *P* = 0.029) were positively correlated with ADL.

**TABLE 3:** The correlation indicators were partially correlated with the ADL In the AD group.

Control age and education		Go accuracy	Go omissions	Go errors	Go errors RT	Go RT	Stop accuracy	Failed stop RT	SSD	SSRT	All accuracy	N3_ mean_ amp	N3_ peak_ amp	N3_ peak_ latency	P2_ mean_ amp	P2_ peak_ amp	P2_ peak_ latency
ADL	r	−***0.603***	0.380	***0.544***	***0.573***	0.470	0.166	***0.488***	0.180	***0.478***	−***0.624***	0.205	0.209	0.061	0.101	0.054	−0.152
	*p*	***0.005***	0.098	***0.013***	***0.008***	0.036	0.484	***0.029***	0.447	***0.033***	***0.003***	0.385	0.376	0.797	0.673	0.822	0.523

ADL, activity of daily living; RT, reaction time; SSD, stop-signal delay; SSRT: stop-signal reaction time. N3_mean_amp, mean amplitude of N300; N3_peak_amp, peak amplitude of N300; N3_peak_latency, peak latency of N300. P2_mean_amp, mean amplitude of P200; P2_peak_amp, peak amplitude of P200; P2_peak_latency, peak latency of P200. Values in bold and italics indicate a partial correlation with ADL.

### 3.4 ROC analysis

**FIGURE 5:**
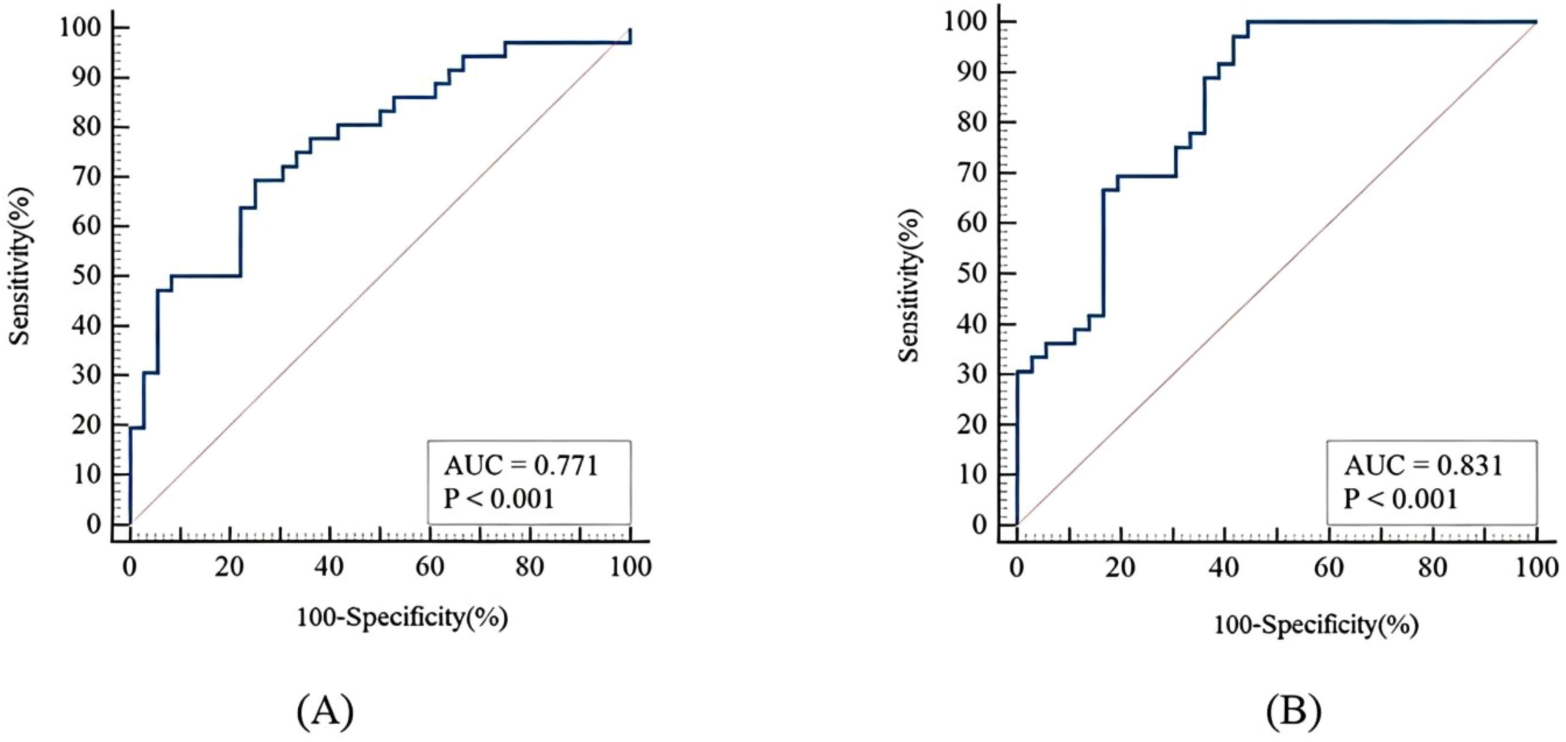
Receiver operating characteristic (ROC) analysis. Combined diagnostic ROC curve **(A)** with different SST-related indexes and combined diagnostic ROC curve **(B)** with different EEG-related indexes between AD and HC groups. Both of them had good combined diagnostic value. AUC, Area Under the Curve.

These different SST-related indicators were combined for diagnosis, and a ROC curve was drawn (Figure 5A). The value of the Area Under the Curve (AUC) was 0.771 (*P*< 0.001). These different EEG-related indicators were combined for diagnosis, and an ROC curve was drawn (Figure 5B) with an AUC of 0.831 (*P*< 0.001). These results indicate that the different SST- and EEG-related indicators have good clinical diagnostic value in combined diagnosis.

## 4 Discussion

In this study, the executive control ability of patients with AD was reflected in the ERPs of the SST with the aim of exploring the relationship between executive control ability and the degree of ADL impairment and other neural mechanisms. First, we found that executive control was significantly reduced in patients with AD compared to HCs, and that the worse the SST performance of patients with AD was, the more severe the ADL impairment. Second, we found that when patients with AD participated in the SST, their EEG data was different from that of HCs. The amplitude of the N300 in patients with AD was reduced, the latency of the N300 was shortened, and the N300 appeared earlier than in HCs. These results indicated that patients with AD had lower excitability in response to stimuli, and indicated that they had signal processing difficulties, which led to worse SST-related responses than that of HCs. We performed ROC analysis on the different SST- and EEG-related indices in the AD and HC groups for the combined diagnosis of AD, both of which had good clinical diagnostic value, revealing alterations in basic brain function in AD and providing new directions for understanding the pathophysiological mechanisms of the disease.

When performing SST tasks, the Go accuracy of AD patients was significantly lower than that of HCs due to impaired executive function (*P*< 0.001). The failed Stop RT in the AD group was higher than that in the HC group, and the difference was statistically significant (*P*< 0.05), indicating that the response inhibition ability of the AD patients was impaired. Differences in SST-related indicators between the two groups indicated that executive control and response inhibition were impaired in patients with AD compared to HCs. After controlling for age and years of education, Go accuracy and all accuracies in the AD group were negatively correlated with ADL. Go errors, Go errors RT, and Failed stop RT were positively correlated with ADL. The results showed a partial correlation between executive function and ADL, with worse executive function being associated with more serious ADL impairment. Conversely, the better the executive function of patients with AD, the better their ADL. The standard deviations of all reaction times in AD patients (Go RT, Go error RT, failed stop RT, SSD, and SSRT) were higher than those of HCs. This indicates that AD patients exhibit poorer overall cognitive stability and sustained attention, with more unstable responses and impaired stability of executive function.

This conclusion is consistent with previous studies: attentional control within executive function gradually declines in the early stage of AD (Baddeley et al., 2001). Deficits in cognitive stability and sustained attention constitute core early features of AD, and behavioral response lability is strongly coupled with multi-component variations in ERPs, which can serve as combined biomarkers for discriminating AD patients from HCs (Horvath et al., 2018). The intra-individual coefficient of variation of reaction time on sustained attention tasks in the AD group is 2.1 times that of the HC group, and lability in executive function is synchronously reflected by markedly elevated intergroup variability in ERPs, quantitatively validating the lability of executive function among AD patients (Tarawneh et al., 2021). Amyloid-beta (Aβ) deposition in the brains of AD patients impairs the frontoparietal cortex and directly undermines the stability of sustained attention; higher AD pathological protein deposition correlates with greater variability in task responses and concurrent deterioration of executive control capacity (McKay et al., 2022).

Patients with mild to moderate AD were selected for our study, and these abnormal SST results and abnormal changes in brain acitivity provided us with new ideas for clinical diagnosis. ROC analysis was performed on the different SST- and EEG-related indicators in the AD and HC groups, and these results showed that these indicators had good clinical diagnostic value in the combined diagnosis. Therefore, this method can be used as an early auxiliary clinical diagnostic tool for AD. ERPs as a biomarker of AD-related neuropathology could extend the spatial knowledge provided by structural and fMRI studies, enhance the understanding of the temporal nature of the communication and connectivity of AD-related neural networks, and provide a reliable basis for interventions to perform control functions in patients with AD, which in the future may help maintain or improve patients’ ADL and reduce caregiver burden.

Inhibition control is a crucial executive function that has been extensively studied using the SST (Boucher et al., 2021). We examined the effect of SST-based executive control on ADL in patients with AD. To date, there have been few reports on the application of SST in patients with AD, which may be due to the decline in cognitive and executive functions of AD patients with AD (Khan et al., 2020; Porsteinsson et al., 2021). When participating in the SST, it is difficult to understand the task and respond, which greatly increases the difficulty of the experiment. In this study, we selected patients with mild AD symptoms to avoid situations in which the subjects could not understand the test. When participating in the SST, the rules of the task were fully explained so that the subjecsts could understand the experimental content to the maximum extent, improve the accuracy of the reaction, ensure the normal conduct of the experiment, and reduce the error caused by patients who could not understand the experimental rules. It is not difficult to predict that this research tool will be increasingly applied to reveal the mechanisms of individual cognitive function and the nature of clinical mental illnesses.

The existing SST paradigm still has shortcomings, and future studies need to develop more ingenious SST variants to investigate the influencing factors or variables related to executive control and response inhibition and solve the problem of the influence of the test strategy on the measurement of the Go RT in the SST. For example, during the process of SST execution, subjects may intentionally wait for the stop signal to appear to maintain a high successful inhibition rate, resulting in deviations in the measurement of the Go RT. Therefore, further improvements to the SST paradigm to eliminate this impact needs to be addressed in the future.

The N300 is a negative-going component with a frontal scalp distribution that peaks approximately 300 ms after the onset of a visual stimulus (Kumar et al., 2021). The N300 reflects the object recognition process (Truman and Mudrik, 2018). Thus far, most articles on the N300 have designated it as an index for “object recognition,” which is considered to be a fast match of indexed visual input with stored semantic knowledge (Schendan and Kutas, 2007; Sitnikova et al., 2008; Sitnikova et al., 2006; Yum et al., 2011). In this study, the stimulus signal of the SST paradigm was a left or right arrow (visual cue). After receiving the visual signal stimulus, the subjects reacted by pressing or not pressing the button. The N300 is well positioned to capture the knowledge of iteration, visual processing, and context-sensitive processes (Porsteinsson et al., 2021). Owing to the decline in the cognitive ability of patients with AD (Fedurco, 2012; Soria Lopez et al., 2019), it is difficult for patients to convert visual signals into stored knowledge (Fedurco, 2012) after receiving visual stimulus signals and quickly make key responses required by the experiment. When visual stimuli are activated, the N300 response has a greater amplitude (Carretié et al., 1997). In this study, the peak N300 value of patients with AD was lower than that in HCs, indicating that patients with AD had lower excitability in response to SST visual signal stimulation. Based on the results of this experiment, the incubation period of the N300 in patients with AD was shortened, and the N300 appeared earlier than in HCs, indicating that patients with AD did not fully process the signals they received. Therefore, we believe that abnormal N300 latency in AD patients reflects delayed stimulus identification during inhibitory processing and reduced cognitive evaluation ability.

P200 reflects early attention allocation to external stimuli and is involved in cognitive processes such as working memory (Lijffijt et al., 2009). Studies have shown that the P200 latency in auditory and olfactory modalities has classification rates of 75.0 and 92.0%, respectively, in distinguishing AD from normal aging (Morgan and Murphy, 2002) . The P200 latency is also able to differentiate progressive mild cognitive impairment (MCI) from stable MCI with a sensitivity of 88.0% and specificity of 77.0% (Missonnier et al., 2007). Additionally, the flash visual evoked potential (FVEP), particularly FVEP-P2, has been considered as one of the electrophysiological biomarkers of AD. AD and MCI patients exhibit delayed FVEP-P2 latency, and the diagnostic accuracy of FVEP-P2 latency is approximately 73.0%, comparable to cerebrospinal fluid (CSF) and neuroimaging accuracy (Arruda et al., 2020) . The results of this study showed no significant differences in P200 amplitude and latency between AD and normal aging groups (*p* > 0.05) (Chang et al., 2014; Kurita et al., 2010), which is consistent with previous studies, indicating that early unconscious attentional processing and initial signal perception functions are relatively preserved in AD patients; However, contrary to previous research, AD patients in this study demonstrated decreased P200 amplitude and prolonged P200 latency (Lister et al., 2016; López Zunini et al., 2016). The mean latency of the P200 peak was prolonged in the AD group (AD = 188.330, HC = 186.940), while both the average amplitude (AD = 1.058, HC = 0.752) and peak amplitude (AD = 1.448, HC = 1.141) showed a slight increase. Whereas the abnormal N300 suggests impairment in late conflict processing and higher-order cognitive functions related to inhibitory control. These findings indicate a cognitive dissociation pattern in AD patients, characterized by preserved early attention but impaired late inhibition. These findings need further validation with a larger sample size.

It is feasible to explore the mechanism of executive function in AD patients using SST-based ERPs, and it may be a sensitive imaging marker in AD patients at early stage. To the best of our knowledge, this study is the first to use SST to study patients with AD. Compared to other neuroimaging methods, EEG has the advantages of being noninvasive, easy to use, and inexpensive (Wang et al., 2019). In the present study, the combination of SST and EEG significantly improved the affordability and compatibility of patients with AD. The combination of SST and ERP can be used as an effective method for the assessment of cognitive functions and executive control in patients with AD, as well as a valuable means for the early diagnosis of AD, which can be used to identify and track the onset and progression of the disease. Our study demonstrated that specific alterations in SST-ERPs can accurately differentiate early AD patients from HCs at the same time, and that we need to pay more attention to ADL in AD patients, actively carry out early intervention activities for AD patients, strengthen the cognitive function training of patients (López-Ortiz et al., 2021; Yu et al., 2021), slow down the decline of ADL in AD patients, improve their quality of life, and relieve pressure on caregivers (Grabher, 2018). In the future, we will further explore the influence and characteristics of pathological changes in AD based on ERPs.

There are some limitations to our study. First, we recruited participants according to the NINCDS-ADRDA criteria. Therefore, the study participants were likely to have AD. A lack of biomarkers may have contributed to this bias. Second, all participants in this study were Han Chinese individuals recruited from Anhui Province, China, with relatively consistent geographical, ethnic, educational and sociodemographic backgrounds. Therefore, the study sample demonstrated a high degree of demographic homogeneity. The number of subjects included in this study was small, and larger sample sizes will be required in the future to reduce potential biases and errors. Third, the research objective of this study was to explore executive function in patients with Alzheimerentsisease, rather than merely the inhibitory control ability reflected by Stop signals. Accordingly, fewer analyses were conducted on Stop-signal-related parameters. In future large-sample studies, we will supplement Stop-signal indicators to enrich the analytical content. Fourth, this was a cross-sectional study, and future longitudinal studies should be designed to confirm these results.

## 5 Conclusion

Compared with HCs, AD patients had lower accuracy and higher error rates when participating in the SST, and the time required to inhibit and control responses was longer, which reflected that AD patients’ executive control ability was lower than that of healthy people. Impaired ADL are associated with executive dysfunction in patients with AD. The worse the executive function, the longer the time required to inhibit the control response and the more severe the ADL impairment. When the executive function is improved and the time required to inhibit the control response is shorter, the degree of ADL impairment is reduced. There were differences in executive control-related EEG indices in patients with AD and HCs; compared with HCs, the N300 amplitude in patients with AD was reduced, the N300 latency was shortened, and the peak appeared earlier. These SST- and EEG-related indices show potential as auxiliary diagnostic tools; however, further validation in larger sample size studies is needed in the future.

## Data Availability

The original contributions presented in the study are included in the article/supplementary material, further inquiries can be directed to the corresponding authors.
